# Long term prognosis in patients with pulmonary hypertension undergoing catheter ablation for supraventricular tachycardia

**DOI:** 10.1038/s41598-021-95508-3

**Published:** 2021-08-10

**Authors:** Hiroyuki Kamada, Junji Kaneyama, Yuko Y. Inoue, Takashi Noda, Nobuhiko Ueda, Kenzaburo Nakajima, Tsukasa Kamakura, Mitsuru Wada, Kohei Ishibashi, Kenichiro Yamagata, Koji Miyamoto, Tatsuo Aoki, Takeshi Ogo, Satoshi Nagase, Takeshi Aiba, Kazuhiro Satomi, Kengo Kusano

**Affiliations:** 1 Department of Cardiovascular Medicine, National Cerebral and Cardiovascular Center, 6-1 Kishibe Shin-machi, Suita, Osaka 564-8565 Japan; 2 Department of Cardiology, Saitama Sekishinkai Hospital, Saitama, Japan; 3 Department of Cardiology, Tokyo Medical University, Tokyo, Japan

**Keywords:** Cardiology, Medical research

## Abstract

Various forms of supraventricular tachycardia (SVT) occur in patients with severe pulmonary hypertension (PH). Despite the high efficacy of radiofrequency catheter ablation (RFCA) for SVT, insufficient data exist regarding patients with PH. Thirty SVTs in 23 PH patients (age 47 [35–60] years; mean pulmonary artery pressure 44 [32–50] mmHg) were analyzed. Procedural success rate, short- and long-term clinical outcomes, were evaluated during a median follow-up of 5.1 years. Single-procedure success rate was 83%; 94% (17/18) in typical atrial flutter, 73% (8/11) in atrial tachycardia (AT), and 100% (1/1) in atrioventricular nodal reentrant tachycardia. Antiarrhythmic drugs, serum brain natriuretic peptide levels and number of hospitalizations significantly decreased after RFCA than that before (*p* = 0.002, 0.04, and 0.002, respectively). Four patients had several procedures. After last RFCA, 12 patients had SVT and 8 patients died. Kaplan–Meier curves showed that patients with SVT after the last RFCA had a lower survival rate compared to those without (*p* = 0.0297). Multivariate analysis identified any SVT after the last RFCA as significant risk factor of mortality (hazard ratio: 9.31; *p* = 0.016). RFCA for SVT in patients with PH is feasible and effective in the short-term, but SVT is common during long-term follow-up and associated with lower survival.

## Introduction

Pulmonary hypertension (PH) is a progressive disease characterized by vascular proliferation and remodeling in the pulmonary vasculature. In recent years, various treatments have been introduced that have improved the prognosis of patients with PH^1,2^. Despite these therapies, however, patients with PH—including those resistant to therapies—still have a high mortality rate due to right ventricular failure^3,4^.

Supraventricular tachycardia (SVT) is an increasingly common clinical problem in patients with PH, and various forms of SVT can occur in these patients. Remodeling of the right atrium in response to longstanding pressure- and volume-overload appears to generate an underlying arrhythmogenic substrate^5,6^. Recent studies suggest that SVT is an independent prognostic factor in patients with PH^7,8^. Thus, from a prognostic perspective it may be worthwhile to maintain a stable sinus rhythm. However, antiarrhythmic drug (AAD) therapy may not be a feasible option because of their negative inotropic properties and interaction with therapeutic drugs for PH^9^. Despite the high efficacy of radiofrequency catheter ablation (RFCA) to treat SVT in healthy subjects, limited data are available regarding the efficacy and safety of RFCA for patients with PH.

The current study presents short- and long-term data from a single-center series of patients with PH undergoing RFCA for SVT and explores the influence of procedural and clinical variables on clinical outcomes.

## Methods

### Eligibility criteria

This was a retrospective single study based on a tertiary center. We enrolled 23 consecutive patients with pre-capillary PH undergoing RFCA for a history of sustained and/or recurrent SVT at the National Cerebral and Cardiovascular Center between January 1998 and October 2019. SVT included atrial flutter (AFL), atrial tachycardia (AT), and atrioventricular nodal reentrant tachycardia (AVNRT); no patients with SVT from left atrium or atrial fibrillation (AF) underwent RFCA in this study. We excluded patients with PH secondary to left-sided heart disease (European Society of Cardiology [ESC]/European Respiratory Society [ERS] Clinical Group 2; pulmonary artery wedge pressure > 15 mmHg; n = 8). PH was defined as mean pulmonary arterial pressure ≥ 25 mmHg at rest, before electrophysiological study (EPS)^10^. All data on pulmonary pressures were obtained from right heart catheterization except for one patient with PH, due to inoperable Eisenmenger syndrome. Data were extracted from medical records and procedure notes. The study was approved by the National Cerebral and Cardiovascular Center ethics review board (M26-148), and we applied Opt-out method to obtain informed consent. The study complied with the Declaration of Helsinki.

### Cardiac electrophysiological study and catheter ablation

If SVT was not present at the beginning of the EPS, induction of SVT was attempted with programmed atrial stimulation from high in the right atrium or coronary sinus, as detailed in the Data Supplement. A three-dimensional (3D) activation sequence map of the right atrium during SVT was constructed in 16 patients (70%). Focal AT (FAT), intra-atrial reentrant tachycardia (IART), and AVNRT were ablated at the earliest lesion, critical isthmus, and slow pathway, respectively. Procedure time was defined as the duration from the puncture to removal of the sheaths.

### Acute procedure outcome measures

Targeted SVT included clinical and non-clinical SVT, except AF, occurring during EPS and RFCA. Acute success or failure were defined as termination of an SVT circuit during application of radiofrequency energy, or no subsequent inducibility by programmed stimulation. Complete success of RFCA was defined as a procedure in which all targeted SVT circuits were terminated, and partial success was defined as a procedure in which one or more targeted SVT circuits were acutely terminated, but not all targeted SVT circuits were ablated, or SVT was unmappable because of unmappable or non-sustained AT.

The safety outcome of RFCA was assessed by the occurrence of complications. The definition of a complication included any event that was causally related to the RFCA and occurred within 24 h of the procedure.

### Short- and long-term clinical outcomes

All patients were evaluated at our center every 1–3 months using a 12-lead electrocardiogram, 24-h Holter monitor, or portable electrocardiograph when patients complained of palpitations. Patients whose symptoms recurred were evaluated immediately. SVT and complications after RFCA were confirmed by medical records and the definition of SVT was arrhythmia lasting for > 30 s.

Three measures were used to evaluate RFCA efficacy in relation to short-term outcomes, including (1) the number of AADs administered on admission and discharge following RFCA, (2) serum brain natriuretic peptide (BNP) levels one month before and after RFCA, and (3) the number of admissions associated with heart failure or arrhythmia within the 1-year period before and after RFCA.

The long-term outcome of a patient was defined as any SVT and all-cause mortality after RFCA. SVT after the RFCA procedure included the recurrence of targeted SVT and new onset of any type of SVT, including AF or another SVT from both atria not targeted during the RFCA, and were divided into long-term SVT (n = 12) and no long-term SVT group (n = 11).

### Statistical analysis

Continuous data were represented as median and interquartile range, and categorical variables as number (percentage). Continuous variables were compared between two groups using non-parametric Mann–Whitney U test, and categorical data were compared using the chi-squared test or Fisher’s exact test where appropriate. According to the distribution of the data sets, the statistical differences between pre-ablation and post-ablation values were determined by two-sided paired t-test, Wilcoxon signed-rank sum test, or McNemar test. Kaplan–Meier survival curves were used to estimate the cumulative incidence of SVT and the survival of patients by the log-rank test. Univariate and multivariate Cox proportional regression analyses were performed to identify risk factors of mortality. Results of the analysis were presented as hazard ratios (HRs) and 95% confidence intervals (CIs). Differences with an associated *P* value < 0.05 were considered statistically significant. All calculations were performed using JMP Pro 14 (SAS Institute, Tokyo, Japan).

## Results

### Patient characteristics

**Figure 1:**
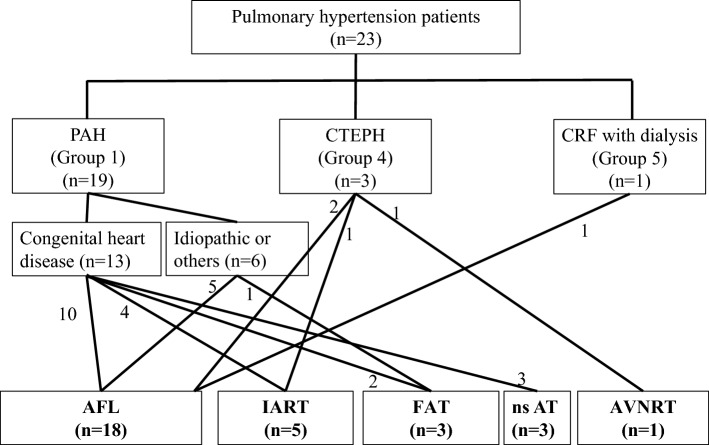
Study population and diagnosis. AFL: atrial flutter, AT: atrial tachycardia, AVNRT: atrioventricular nodal reentrant tachycardia, CRF: chronic renal failure, CTEPH: chronic thromboembolic pulmonary hypertension, FAT: focal atrial tachycardia, IART: intra-atrial reentrant tachycardia, ns: non-sustained, PAH: pulmonary arterial hypertension.

A total of 23 PH patients (mean PA pressure, 44 [32–50] mmHg) with 30 SVTs were enrolled; 19 with pulmonary arterial hypertension (PAH), 3 with chronic thromboembolic PH (CTEPH), and 1 with hemodialysis (Fig. 1). Table 1 shows the baseline characteristics of our cohort. There were 15 females and 8 males with a median age of 47 (35–60) years. The PAH included 13 patients with congenital heart disease (CHD), including atrial septal defect (ASD), ventricular septal defect (VSD), tetralogy of Fallot (TOF), atrioventricular septal defect (AVSD) and anomalous pulmonary venous return. Twelve of 13 patients with CHD had prior cardiac surgical intervention, 2 patients (numbers 10 and 16) had previously received a pacemaker for sick sinus syndrome, and none of CTEPH patients had inferior vena cava filters (Data Supplement and Supplemental Table 1).

**Table 1:** Baseline characteristics according to the presence or absence of SVT after the last RFCA procedure.

	All	Long-term SVT (n = 12)	No long-term SVT (n = 11)	*p* value
Age, years	47 (35–60)	44 (35–56)	51 (35–66)	0.22
Female sex	15 (65%)	8 (67%)	7 (64%)	1.00
PAH	19 (83%)	12 (100%)	7 (64%)	0.0373
CHD	13 (57%)	8 (67%)	5 (45%)	0.30
CTEPH	3 (13%)	0 (0%)	3 (27%)	0.093
CRF with dialysis	1 (4%)	0 (0%)	1 (9%)	0.48
Intravenous PGI2	8 (35%)	5 (42%)	3 (27%)	0.67
Number of AADs	1 (0–2)	2 (0.3–2.8)	1 (0–2)	0.22
BNP level, pg/mL	178 (60–254)	156 (97–397)	218 (54–253)	0.70
Hospitalizations before RFCA, times/year	1 (0–1)	1 (0–1)	0 (0–1)	0.19
Right heart catheterization				
Mean PAP, mmHg	44 (32–50)	43 (34–50)	44 (28–48)	0.67
RAP, mmHg	8 (5–13)	10 (8–19)	6 (3–11)	0.14
CO, L/min	3.8 (2.4–4.4)	2.7 (2.2–4.2)	4.3 (2.8–4.8)	0.19
CI, L/min/m^2^	2.5 (1.6–3.1)	1.7 (1.6–2.9)	2.7 (2.0–3.2)	0.42
SvO_2_, %	67 (57–80)	68 (63–78)	63 (42–69)	0.18
PCWP, mmHg	9 (6–15)	11 (7–18)	9 (4–12)	0.28
PVR, dyne·sec/cm^5^	733 (628–1204)	987 (696–1364)	650 (521–1049)	0.14
RFCA				
AFL	18 (78%)	11 (92%)	7 (64%)	0.16
AT*	8 (48%)	4 (33%)	4 (36%)	1.00
AVNRT	1 (4%)	0 (0%)	1 (9%)	0.48

AAD antiarrhythmic drug, AF: atrial fibrillation, AFL: common atrial flutter, AT: atrial tachycardia, AVNRT: atrioventricular nodal reentrant tachycardia, BNP: brain natriuretic peptide, CHD: congenital heart disease, CI: cardiac index, CO: cardiac output, CRF: chronic renal failure, CTEPH: chronic thromboembolic pulmonary hypertension, PAH: pulmonary arterial hypertension, PAP: pulmonary artery pressure, PCWP: pulmonary capillary wedge pressure, PG: prostaglandin, PVR: pulmonary vascular resistance, RAP: right atrium pressure, RFCA: radiofrequency catheter ablation, SvO_2_: mixed venous oxygen saturation, SVT: supraventricular tachycardia.

*AT did not include non-sustained AT in this table.

### Electrophysiological study and ablation

**Figure 2:**
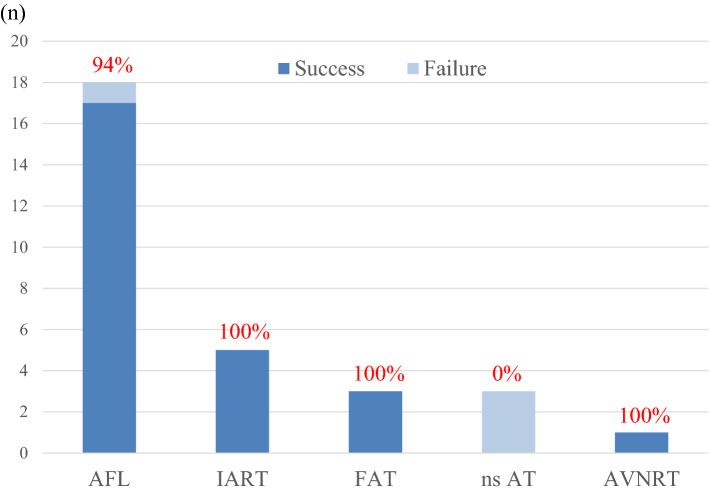
Changes before and after the first RFCA procedure. (**A**) Number of AADs administered 1 month before and after the first RFCA. (**B**) Serum BNP levels 1 month before and after the first RFCA. (**C**) Number of hospitalizations due to heart failure or arrhythmia during 1 year before and after the first RFCA. AAD: antiarrhythmic drug, BNP: brain natriuretic peptide, RFCA: radiofrequency catheter ablation.

Supplemental Table 2 shows the characteristics of the procedures. At the time of the first procedure, the arrhythmia diagnoses were AFL in 18, IART in 5, FAT in 3, and AVNRT in 1 patient. Multiple distinct atrial arrhythmias were present in 4 patients (for example, AFL and IART or FAT; Supplemental Table 2). CHD was present in 10 of 18 patients with AFL, and in 6 of 8 patients with AT. Representative cases with AFL and AT are illustrated in Supplemental Figures. 1 and 2, respectively. At the first procedure, cavo tricuspid isthmus (CTI) ablation was performed in 18 patients, AT ablation in 10 patients, and slow pathway ablation in 1 patient. The rate of complete success following a single procedure was 83% (19/23 patients) and the partial success rate was 17% (4/23 patients). The acute complete success rate following the first procedure was 87% (26/30 SVT): 94% (17/18) in patients with AFL, 72% (8/11) in patients with AT, and 100% (1/1) in a patient with AVNRT (Fig. 2). The partially successful procedures consisted of patients with AFL (n = 1) and unmappable non-sustained (ns) AT (n = 3).

**Table 2:** Cox regression analysis of risk factors for mortality.

	Univariate analysis		Multivariate analysis	
	Hazard ratio (95% CI)	*p* value	Hazard ratio (95% CI)	*p* value
Age, yrs	0.99 (0.95–1.04)	0.71	1.02 (0.96–1.10)	0.51
Sex, Female	2.14 (0.43–10.68)	0.32	4.13 (0.58–29.40)	0.12
CHD	0.82 (0.19–3.53)	0.79		
Intravenous PGI2	**4.23 (0.99–18.00)**	**0.047**	5.10 (0.74–35.00)	0.07
Number of AADs	1.49 (0.81–2.76)	0.19		
BNP level, pg/mL	1.00 (0.99–1.01)	0.12		
Hospitalizations before RFCA, times/year	3.22 (0.66–23.46)	0.15		
Mean PAP, mmHg	0.99 (0.93–1.04)	0.73		
RAP, mmHg	0.98 (0.87–1.08)	0.67		
CO, L/min	0.71 (0.35–1.42)	0.33		
CI, L/min/m^2^	0.67 (0.24–1.58)	0.37		
SvO_2_, %	1.01 (0.96–1.09)	0.66		
PCWP, mmHg	0.93 (0.82–1.06)	0.28		
PVR, dyne·sec/cm^5^	1.00 (0.99–1.00)	0.89		
Any SVT after last RFCA	**5.19 (1.01–26.65)**	**0.031**	**9.31 (1.19–73.01)**	**0.016**

AAD antiarrhythmic drug, BNP: brain natriuretic peptide, CHD: congenital heart disease, CI: cardiac index, CO: cardiac output, PAP: pulmonary artery pressure, PCWP: pulmonary capillary wedge pressure, PG: prostaglandin, PVR: pulmonary vascular resistance, RAP: right atrium pressure, RFCA: radiofrequency catheter ablation, SvO_2_: mixed venous oxygen saturation, SVT: supraventricular tachycardia.

### Safety

The average procedure time was 195 (144–295) minutes (Supplemental Table 2). There was no associated heart failure, or requirement for intubation, inotropic therapy, or cardiac mechanical support. The only complication was bradycardia due to a junctional rhythm, which developed the same day of the procedure in a 22-year-old man who had successful ablation of persistent IART that sustained for 3.2 years (Case 2). Because of exacerbation of heart failure by junctional rhythm and sinus arrest that emerged after RFCA, a permanent pacemaker had been implanted after 8 days, resulting in improvement of the heart failure.

### Short-term effectiveness of ablation

**Figure 3:**
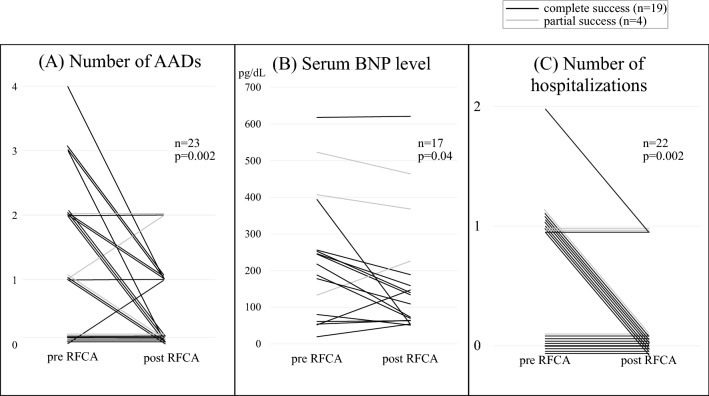
Acute success rate of radiofrequency catheter ablation for supraventricular tachycardia. AFL: atrial flutter, AT: atrial tachycardia, AVNRT: atrioventricular nodal reentrant tachycardia, FAT: focal atrial tachycardia, IART: intra-atrial reentrant tachycardia, ns: non-sustained.

Twelve of 16 patients were able to decrease the number of AADs administered, and the number of AADs decreased from 1.1 to 0.5 drugs per patient after the first RFCA procedure (n = 23, *p* = 0.002, Fig. 3A). Serum BNP levels significantly improved when comparing pre-RFCA (sample before RFCA: 6 [4–13] days) levels to those after RFCA (sample after RFCA: 35 [25—58] days); there was a reduction from 231 pg/dl to 176 pg/dl (n = 17, *p* = 0.04, Fig. 3B). Although serum BNP levels decreased significantly in patients with completely successful RFCA (n = 14, *p* = 0.03, 204 pg/dl vs. 138 pg/dl), the levels did not reduce in patients with partially successful RFCA (n = 3, *p* = 0.98, 354 pg/dl vs. 353 pg/dl). Moreover, the average number of hospitalizations within 1 year before and after RFCA also decreased significantly from 0.6 to 0.1 episodes per patient (n = 22, *p* = 0.002, Fig. 3C).

### Long-term follow-up

We performed a second procedure in 4 patients (in 3 patients due to recurrence of targeted SVT in the first procedure and in 1 patient due to the onset of new SVT), and a third procedure in 1 patient who had recurrence of targeted SVT in the second procedure; all procedures were a complete success in the end (Supplemental Table 2). During a median follow-up of 5.1 year (2.1–9.1) after the last procedure, SVTs occurred in 12 patients (9 AT and 3 AF), of which recurrence of the targeted SVT was seen in 2 patients (both with AT) and new onset of SVT was seen in the remaining 10 patients (7 AT and 3 AF; Supplemental Table 2). Rate control was performed with verapamil or digoxin in the 2 patients with symptomatic non-sustained AT.

**Figure 4:**
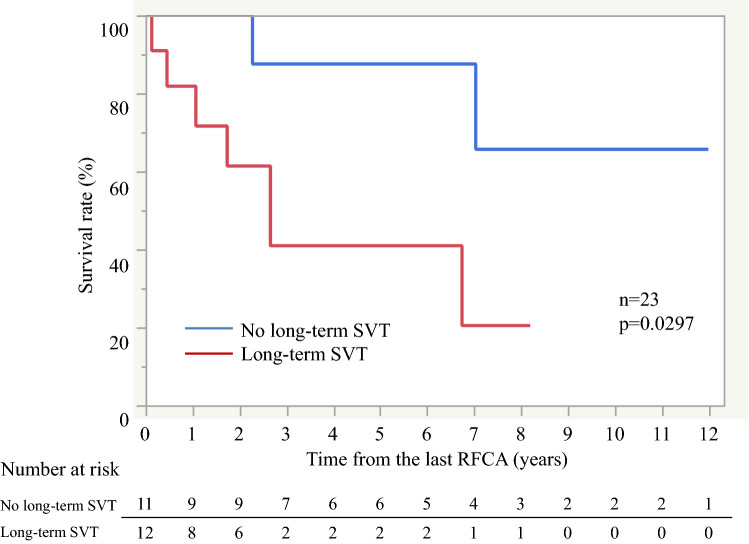
Kaplan-Myer survival curves between with and without SVT after the last RFCA. RFCA: radiofrequency catheter ablation, SVT: supraventricular tachycardia.

There was no difference in the baseline characteristics between patients with and without any SVT after the last RFCA procedure (Table 1). Overall, there were 8 deaths among 23 patients during follow-up, including 6 patients due to worsening of right cardiac failure, 1 patient who experienced sudden death, and 1 patient who died of unknown cause. The 1-, 3-, 5-, and 10-year survival rates after the first procedure were 100%, 80%, 74%, and 43%, respectively (Supplemental Fig. 3A). In the long-term, Kaplan–Meier survival curves showed that patients with any SVT after the last RFCA had a lower survival rate compared to those without (*p* = 0.0297, Fig. 4). There was same trend for higher all-cause mortality with SVT after the last RFCA in only patients with PAH, though the difference was not statistically significant (*p* = 0.10, Supplemental Fig. 3B). Univariate analysis revealed that intravenous PGI2 and any SVT after last RFCA were significant risk factors for mortality (intravenous PGI2 HR 4.23, 95%CI 0.99–18.00, *p* = 0.047; any SVT after last RFCA HR 5.19, 95%CI 1.01–26.65, *p* = 0.031). Multivariate analysis revealed that any SVT after last RFCA was an independent risk factor for mortality (HR 9.31, 95%CI 1.19–73.01, *p* = 0.016, Table 2).

## Discussion

### Main findings

This study showed that (1) the RFCA procedure for SVT in patients with PH is successful and safe, and non-sustained and unmappable AT results in RFCA failure; (2) in the short-term, RFCA improves quality of life (QOL) such as decreasing the number of AADs taken, BNP levels, and the number of hospitalizations; and (3) several new-onset SVTs occur over the long-term, and overall prognosis is not good, especially in those where all SVTs were not eliminated. Our findings are particularly important because, to our knowledge, this is the first report to demonstrate that the any SVTs after RFCA are significantly associated to mortality in patients with PH during long-term follow-up after RFCA.

### Safety

Catheter ablation of SVT in patients with PH might be challenging in clinical practice, because mortality following heart catheterization for PH has been reported to be high^11^. A more recent study reported the mortality to be lower in a special facility for patients with PH^12^. Warren et al. identified predictors of catheterization-related complications in the PH population. Their predictors included age < 2 years, use of general anesthesia, need for intervention, and mean right atrial pressure^13^. Risk factors for morbidity and mortality in non-cardiac surgery may also be useful predictors^14^. Although a patient with high right atrial pressure and mean PAP corresponded to general anesthesia in this study, none faced clinical deterioration as a result of RFCA.

The frequency of complications associated with catheter ablation for SVT in non-PH patients is not high^15^. In the few reports including small numbers of patients with PH, the complication rate of RFCA for SVT was 0–14% (Table 3)^5,6,16,17^. Bradfield et al. reported the exacerbation of right heart failure and death from lead dislodgement after AVNRT ablation to be potential complications. One patient in our study experienced bradycardia after RFCA for persistent IART of 3.2 years duration, and it was treated with permanent pacemaker for the alleviation of heart failure 8 days later, because the sudden onset of bradycardia may cause hemodynamic deterioration in patients with PAH^18^. One should remain aware of sick sinus syndrome after RFCA in patients with PH as well as those with a long duration of SVT^19^.

**Table 3:** Previous literature about RFCA for SVT in patients with pulmonary hypertension.

Author	ESC/ERS PH Clinical group	Mean PA pressure (mmHg)	Target SVT	Success rate	Complications
Tongers et al.^5^	1, 4 (n = unknown)	50 ± 10	AVNRT: 3	100% (3/3)	0% (0/8)
			AFL: 5	100% (5/5)	
Bradfield et al.^6^	1 (n = 11),	99 ± 35	AFL: 12	86% (12/14)	14% (2/14)
	4 (n = 1)	(systolic PA)			Right heart failure: 1
					Death: 1
Showkathali et al.^16^	1 (n = 11),	54.5 ± 11.1	AFL: 22	100% (22/22)	0% (0/22)
	4 (n = 11)				
Luesebrink et al.^17^	1 (n = 3), 2 (n = 32), 4 (n = 3)	36.3 ± 13.5	AFL	100% (38/38)	0% (0/38)
Bandorski et al. 20)	1 (n = 14), 2 (n = 23), 3 (n = 4), 4 (n = 8), 5 (n = 2), other (n = 4)	Unknown	AFL: 14	86% (12/14)	Unknown
			AT: 11	18% (2/11)	
			IART: 2	100% (2/2)	
			AVNRT: 4	100% (4/4)	

AFL: common atrial flutter, AT: atrial tachycardia, AVNRT: atrioventricular nodal reentrant tachycardia, ERS: European Respiratory Society, ESC: European Society of Cardiology, IART: intra-atrial reentrant tachycardia, PA: pulmonary arterial, PH: pulmonary hypertension, RFCA: radiofrequency catheter ablation, SD: standard deviation, SVT: supraventricular tachycardia.

### Short-term efficacy of RFCA

Our study population comprised of patients mostly with AAD-resistant symptomatic SVT. Recent guidelines recommend therapy for SVT with AADs without negative inotropic effects such as amiodarone, although amiodarone can interact with PAH-targeted therapies and may cause life-threatening thyroid dysfunction^8,9^; furthermore, specific data regarding efficacy are lacking.

In previous studies about RFCA for SVT in patients with PH, the acute success rate of RFCA for typical AFL was 86–100%^5,6,15,16,20^, which is in accordance with our study results. Anatomical remodeling may increase RF lesions to obtain bidirectional isthmus block, but does not appear to have an impact on the acute success rate of RFCA for AFL. Compared with AFL, less research has been done on RFCA for AVNRT and AT in patients with PH. Tongers et al. reported the acute success rate of RFCA for AVNRT to be 100% in patients with PH^5^, which is in accordance with our study results. Bandorski et al. reported the acute success rate of RFCA for AT in patients with PH to be 31% (4/13 patients)^20^. Although the success rate of IART was 100% (2/2), the success rate for other focal or ns ATs was only 18% (2/11) due to multifocal tachycardia, or inability to place the catheter into a dilated right atrium. On the other hand, our results showed a higher acute success rate of RFCA for AT at 73% (8/11; 5/11 IART and 3/3 FAT). The reason for failure was unmappable non-sustained ATs of unknown mechanism, and all the mappable ATs were successfully ablated in our study. Two reasons may explain these different results. First, the past study mainly included patients with PH associated with left-sided heart disease (42%)^20^ and multifocal complex substrates AT (57%) including the left atrium. Our study consisted of PH patients who underwent SVT ablation in only right atrium because hemodynamic deterioration after AF RFCA with pulmonary vein isolation has been reported in patients with PH^21^. In addition, this study mainly included CHD-related PH patients (57%), although PH multicenter registry showed that there was no significant difference in prognosis between patients with idiopathic PAH and CHD^22^. Second, the introduction of a 3D mapping system may have contributed to the success rate.

Bradfield et al. reported an improvement in serum BNP levels and systolic pulmonary arterial pressures after RFCA for AFL in patients with PH^6^. Our research revealed that RFCA for SVT decreased not only the number of AADs administered and serum BNP levels, but also hospital admission due to heart failure within 1 year of RFCA.

### Long-term efficacy of RFCA

SVT in patients with PH may not only represent a simple marker of prognosis but may also be a cause of clinical deterioration. Recent prospective observational studies have suggested that male sex, older age, higher World Health Organization functional class (WHO FC), and lower SvO_2_ at baseline are strong predictors of worse survival in patients with PAH^23,24^. Permanent AF is also reported to be an independent prognostic factor in patients with PH^7^. In our study, patients with any SVT after the last RFCA procedure had similar baseline characteristics but a worse prognosis compared with those without. Patients with PH in our study had similar survival rates compared to those in a Japanese multicenter PH registry (3-year survival; 80% vs. 88%)^25^ and patients with PH without any SVT after RFCA had a higher survival rate compared to those with (3-year survival; 88% vs. 41%). This may indicate that treatment of SVT by RFCA could avoid clinical deterioration and improve the prognosis of patients with PH. Further prospective studies are needed to investigate whether the survival rate improves if all SVTs are eliminated by treatment such as prophylaxis with AADs, electric cardioversion, or RFCA.

## Limitations

This study has few limitations. First, our study may have been subjected to selection bias because it was a single-center study, and indications for RFCA and hospitalization for SVT were based on the clinical situation of the patient and the judgment of specialists. Second, we included data from an old era before the use of current treatments. The survival rate in this study might be worse than the actual survival rate. Third, the association between SVT after ablation and mortality might not necessarily imply a causal relationship and this retrospective study did not exclude the possibility that the progression of the underlying PH disease itself caused long-term SVT and poor prognosis. Finally, the number of this study was relatively small and the study population was heterogeneous because PH is relatively rare. Information bias also could be an issue because of the missing data resulting from the retrospective nature of this study.

## Conclusion

RFCA for SVT in patients with PH is a safe and effective therapy in the short-term, but recurrence of targeted and non-targeted SVT was common and associated with higher all-cause mortality in the long-term. Our data suggest that maintenance of a stable sinus rhythm after RFCA should be considered an important treatment goal in patients with PH.

## Supplementary Information

Supplementary Information 1.

Supplementary Information 2.
