# Supplementary material for: Long term prognosis in patients with pulmonary hypertension undergoing catheter ablation for supraventricular tachycardia
Source: Sci Rep. 2021 Aug 10;11:16176. doi: 10.1038/s41598-021-95508-3 (PMC8355112; doi:10.1038/s41598-021-95508-3)
Supplement: Supplementary file 1 — Supplementary Information 1. [file 41598_2021_95508_MOESM1_ESM.docx]

**SUPPLEMENTAL MATERIALS**

**Supplemental Methods**

## **Cardiac electrophysiological study and catheter ablation**

The diagnosis of supraventricular tachycardia (SVT) was always confirmed by two additional cardiologists with special expertise in cardiac arrhythmias. After obtaining written informed consent, electrophysiological studies (EPS) were performed under local (n=22) and general (n=1) anesthesia. We used sedative drugs in five patients (midazolam, propofol or thiopental). Nitric oxide was not used during any procedures.

Catheters were introduced and positioned in the right atrium, right ventricular apex, coronary sinus, and His-bundle region. Three-dimensional (3D) mapping systems from CARTO (Biosense Webster Inc., Irvine, CA, USA), EnSite (Abbott, Chicago, IL, USA), or RHYTHMIA (Boston Scientific, Marlborough, MA, USA) were used at the operator’s discretion. We also measured the post-pacing interval at the right atrium, and the paced site was considered within the tachycardia circuit if the post-pacing interval minus the tachycardia cycle length was <30 ms when pacing was 20-30 ms faster than the tachycardia cycle length.

Based on 3D mapping, four different types of SVT were distinguished: (1) typical atrial flutter (AFL): a single counter-clockwise, cavo tricuspid isthmus (CTI)-dependent macro reentrant circuit; (2) intra-atrial reentrant tachycardia (IART): a macro reentrant tachycardia involving scar tissue or suture lines; (3) focal atrial tachycardia (FAT): electrical activation originating from a small, circumscribed region from where it expands to the remainder of the atrium; and (4) atrioventricular nodal reentrant tachycardia (AVNRT): a tachycardia resulting from reentry in the region of the atrioventricular (AV) junction due to the dual AV node pathway.

The ablation catheter varied based on physician preference. Standard ablation was performed using a variety of power- and temperature-controlled catheters (7 F, 4-mm or 8-mm electrode). Power was limited to 30 W and, in the case of temperature-controlled catheters, tip temperature was limited to 55°C. During recent radiofrequency catheter ablation (RFCA), a power-controlled (30 W), irrigated ablation with a closed coolant system was used. Power was delivered for up to 30 s per lesion.

At the commencement of the first procedure, 13 patients presented with SVT (11 AFL patients, 2 atrial tachycardia [AT] patients). Five of 10 patients in whom SVT was not present at the start of the first procedure showed sustained SVT following induction (typical AVNRT in 1 patient, AT in 4 patients). In patients with typical AFL induced during an EPS (n=13), or clinically diagnosed (n=5) by a sawtooth-like pattern on 12-lead electrocardiogram in leads II, III and aVF, CTI ablation was performed. AT consisted of 4 IART and 3 FAT.

The endpoint of ablation was the bidirectional block of CTI in AFL, tachycardia termination or no-inducibility in AT, and disappearance of jump-up or jump-up plus one echo in typical AVNRT.

**Supplemental Results**

**Patient characteristics**

Symptoms related to SVT before the initial RFCA were palpitations in 10 patients (44%), pre-syncope or syncope in 5 patients (22%), right heart failure in 8 patients (35%), and asymptomatic hypotension in 2 patients (9%). Verapamil induced hypotension and right heart failure in patients 6, 8, and 13.

Due to the need to control the ventricular response and/or convert to sinus rhythm in refractory SVT, patients were administered a median of 2 (1 - 3) kinds of antiarrhythmic drug (class I: 10 patients [44%], class II: 2 patients [9%], class III: 1 patient [4%], class IV: 12 patients [52%], and digoxin: 15 patients [65%]).

## **Abbreviations and Acronyms**

3D = three-dimensional

AAD = antiarrhythmic drug

AF = atrial fibrillation

AFL = atrial flutter

ASD = atrial septal defect

AT = atrial tachycardia

AVNRT = atrioventricular nodal reentrant tachycardia

AVSD = atrioventricular septal defect

BNP = brain natriuretic peptide

CHD = congenital heart disease

CI = confidence interval

CTEPH = chronic thromboembolic pulmonary hypertension

CTI = cavo tricuspid isthmus

EPS = electrophysiological study

FAT = focal atrial tachycardia

HR = hazard ratio

IART = intra-atrial reentrant tachycardia

PH = pulmonary hypertension

RFCA = radiofrequency catheter ablation

SVT = supraventricular tachycardia

TOF = tetralogy of Fallot

VSD = ventricular septal defect

**Supplemental Figure 1: Typical AFL and focal AT in a patient with AVSD repair (Case 9)**

1. Twelve-lead electrocardiogram.
2. Three-dimensional activation map of the right atrium during AFL and focal AT.

The left activation map represents counter clockwise-rotated AFL around the tricuspid valve. Post-pacing intervals at points A, B, C, and D are the same duration. An activation map showing focal AT originating from the right atrium. The black arrow represents the earliest site of focal AT. AT stopped during radiofrequency current to this site.

AFL: atrial flutter, AT: atrial tachycardia, AVSD: atrioventricular septal defect, PA: posteroanterior view.

**Supplemental Figure 2: AFL and incisional AT in patient with CTEPH (Case 21)**

1. Twelve-lead electrocardiogram.
2. Three-dimensional activation map of the right atrium during Incisional AT and AFL.

This patient underwent PEA for CTEPH. Double potential represents the incision line of the right atrium during PEA. The RAO view shows the counter-clockwise rotated AT around the incision line. Post-pacing intervals at points A, B, and C are no longer than 30 ms.

AP: anteroposterior view, AFL: atrial flutter, AT: atrial tachycardia, CTEPH: chronic thromboembolic pulmonary hypertension, RAO: right anterior oblique view, PEA: pulmonary endarterectomy.

**Supplemental Figure 3A: Kaplan-Meier survival curve in all PH patients after the first RFCA**

**Supplemental Figure 3B: Kaplan-Meier survival curves in patients with PAH between with and without SVT after the last RFCA**

PAH: pulmonary arterial hypertension, PH: pulmonary hypertension, RFCA: radiofrequency catheter ablation, SVT: supraventricular tachycardia

**Supplemental Table 1: Characteristics of study population**

| Pt no. | Sex | Age (y) | PH classification | Mean PAP  (mmHg) | RAP  (mmHg) | CO/CI | SvO_2_  (%) | PH and catecholamine drugs | No. of AADs | Symptoms | Time from SVT onset to RFCA (days) |
| --- | --- | --- | --- | --- | --- | --- | --- | --- | --- | --- | --- |
| 1 | M | 31 | VSD repair | 37 | 8 | -/- | - | none | 4 | Syncope | 6160 |
| 2 | M | 22 | ASD, TAPVC repair | 28 | 6 | 4.7/4.2 | - | none | 1 | Palpitation | 5266 |
| 3 | F | 39 | ASD, PAPVC repair | 56 | 6 | 5.0/3.2 | - | Beraprost | 2 | Heart failure | 574 |
| 4 | M | 70 | IPAH | 45 | 11 | 2.0/1.3 | - | Beraprost, intravenous PGI2 | 2 | Palpitation | 49 |
| 5 | F | 35 | ASD repair | 64 | 24 | 1.2/0.8 | 38 | intravenous PGI2, dobutamine | 0 | Heart failure | 59 |
| 6 | F | 39 | ASD repair | 28 | 8 | 2.6/1.6 | - | intravenous PGI2 | 2 | Heart failure | 245 |
| 7 | F | 44 | VSD repair | 56 | 10 | 2.0/1.5 | 66  (O_2_ 3L) | Sildenafil, intravenous PGI2 | 1 | Heart failure | 410 |
| 8 | F | 45 | VSD, PDA repair | 50 | 21 | -/- | 62 | Beraprost, Bosentan | 1 | Heart failure | 120 |
| 9 | M | 17 | AVSD repair | 33 | 12 | -/- | 78 | Beraprost, Dipyridamole, intravenous PGI2 | 0 | None | 105 |
| 10 | M | 60 | ASD, VSD repair | 35 | 20 | -/- | 84 | none | 0 | Heart failure | 1279 |
| 11 | F | 56 | CTEPH | 41 | 1 | 5.2/3.1 | 67 | Beraprost | 0 | Pre-syncope, palpitation | 412 |
| 12 | F | 57 | Portal PAH | 44 | 1 | 4.2/2.8 | 78 | none | 2 | Palpitation | 424 |
| 13 | M | 65 | PPH | 48 | 13 | 4.3/2.5 | 57 | Bosentan | 2 | Pre-syncope, BP↓ | 28 |
| 14 | M | 52 | VSD, Eisemenger | 79 | - | -/- | - | none | 0 | Palpitation | 82 |
| 15 | F | 60 | IPAH | 45 | 19 | 2.3/1.7 | 68 | Bosentan | 3 | Heart failure | 1201 |
| 16 | M | 66 | PAH associated with CRF | 44 | 6 | 4.8/2.8 | 63 | Sildenafil | 1 | Syncope | 87 |
| 17 | F | 37 | TOF repair, residual VSD | 32 | 14 | -/- | - | none | 2 | Palpitation | 118 |
| 18 | F | 47 | PAH (CTD) | 50 | 4 | 2.9/1.8 | 68 | intravenous PGI2 | 3 | Pre-syncope, palpitation | 1372 |
| 19 | F | 25 | TOF repair, residual VSD | 25 | 5 | -/- | - | none | 3 | Palpitation | 76 |
| 20 | F | 34 | IPAH | 41 | 9 | 4.3/3/3 | 45 | intravenous PGI2, dopamine, dobutamine | 3 | palpitation | 27 |
| 21 | F | 69 | CTEPH after PEA | 48 | 8 | 3.1/2.3 | 42 | Riociguat | 1 | BP↓ | 19 |
| 22 | F | 64 | ASD repair | 30 | 3 | 3.3/2.4 | 69 | intravenous PGI2, Macitentan, Tadalafil | 0 | Heart failure | 40 |
| 23 | F | 51 | CTEPH | 25 | 2 | 4.4/3.0 | 72 | none | 0 | palpitation | 439 |

AAD: antiarrhythmic drug, ASD: atrial septal defect, AVSD: atrioventricular septal defect, CI: cardiac index (L/min/m2), CO: cardiac output (L/min), CRF: chronic renal failure, CTD: connective tissue disease, CTEPH: chronic thromboembolic pulmonary hypertension, IPAH: idiopathic pulmonary arterial hypertension, PAH: pulmonary arterial hypertension, PAP: pulmonary artery pressure, PAPVC: partial anomalous pulmonary venous return, PDA: patent ductus arteriosus, PEA: pulmonary endarterectomy, PG: prostaglandin, PH: pulmonary hypertension, PPH: primary pulmonary hypertension, RFCA: radiofrequency catheter ablation, SvO_2_: mixed venous oxygen saturation, SVT: supraventricular tachycardia, TAPVC: total anomalous pulmonary venous return, TOF: tetralogy of Fallot, VSD: ventricular septal defect.

**Supplemental Table 2: Procedures and outcomes**

| Pt. | SVT Duration* | Target SVT | Catheter | 3D-mapping | Procedure time (mins) | Success/Partial success | SVT after procedures | Survived or died (cause) |
| --- | --- | --- | --- | --- | --- | --- | --- | --- |
| 1 | Paroxysmal | AFL | 8 mm | None | 175 | Success | AT | Survived |
| 2-1 | L-S persistent | IART | Unknown | CARTO | 247 | Success | - | - |
| 2-2 | Paroxysmal | AFL | 4 mm irrigated | CARTO | 198 | Success | None | Survived |
| 3 | Paroxysmal | AFL | Med Cosio XL | None | 104 | Success | None | Survived |
| 4 | Paroxysmal | AFL | 4 mm | CARTO | 193 | Success | None | Died (right cardiac failure) |
| 5 | Persistent | IART | 4 mm | CARTO | 180 | Success | None | Survived |
| 6 | Paroxysmal | AFL, ns AT | 4 mm | CARTO | 315 | Partial success (ns AT) | AT | Died (right cardiac failure) |
| 7-1 | Paroxysmal | IART, AFL | 4 mm, 8 mm | CARTO | 158 | Partial success (AFL) | - | - |
| 7-2 | Paroxysmal | IART, AFL | 4 mm, 8 mm | CARTO | 138 | Success | - | - |
| 7-3 | Paroxysmal | IART | 4 mm irrigated | CARTO | 230 | Success | AT | Died (right cardiac failure) |
| 8 | Persistent | AFL, IART, ns AT | 8 mm | CARTO | 192 | Partial success (ns AT) | AT | Died (right cardiac failure) |
| 9 | Paroxysmal | AFL, FAT | 4 mm | CARTO | 352 | Success | AF | Died (right cardiac failure) |
| 10 | Paroxysmal | AFL | 4 mm | CARTO | 300 | Success | AT | Survived |
| 11 | Paroxysmal | AVNRT | 4 mm | None | 215 | Success | None | Survived |
| 12 | Paroxysmal | FAT | 4 mm | None | 104 | Success | AT | Died (right cardiac failure) |
| 13 | Paroxysmal | AFL | 4 mm | CARTO | 168 | Success | None | Survived |
| 14 | Paroxysmal | AFL, ns AT | 8 mm | None | 172 | Partial success (ns AT) | AF | Survived |
| 15 | Paroxysmal | AFL | 4 mm, 8 mm | CARTO | 322 | Success | AT | Survived |
| 16 | Paroxysmal | AFL | 8 mm | None | 90 | Success | None | Survived |
| 17 | Paroxysmal | AFL | 4 mm | None | 295 | Success | AT | Survived |
| 18 | Paroxysmal | AFL | 4 mm irrigated | CARTO | 197 | Success | AF | Survived |
| 19-1 | Paroxysmal | FAT | 4 mm | CARTO | 395 | Success | - | - |
| 19-2 | Paroxysmal | FAT | 4 mm | CARTO | 295 | Success | None | Died (sudden death) |
| 20-1 | Persistent | AFL | 4 mm | CARTO | 197 | Success | - | - |
| 20-2 | Persistent | AFL | 4 mm | CARTO | 330 | Success | AT | Died (unknown cause) |
| 21 | Paroxysmal | AFL, IART | 4 mm irrigated | CARTO | 139 | Success | None | Survived |
| 22 | Paroxysmal | AFL | 4 mm irrigated | Ensite | 120 | Success | None | Survived |
| 23 | Paroxysmal | AFL | 4 mm irrigated | Rhythmia | 90 | Success | None | Survived |

AF: atrial fibrillation, AFL: common atrial flutter, AT: atrial tachycardia, AVNRT: atrioventricular nodal reentrant tachycardia, CTI: cavo tricuspid isthmus, FAT: focal atrial tachycardia, IART: intra-atrial reentrant tachycardia, L-S: long-standing, ns: non-sustained.

*Paroxysmal SVT is characterized by self-terminating episodes within 7 days. Persistent SVT lasts longer than 7 days and less than 1 year. Long-standing persistent SVT lasts longer than 1 year.
