# Supplementary material for: Long term prognosis in patients with pulmonary hypertension undergoing catheter ablation for supraventricular tachycardia
Source: Sci Rep. 2021 Aug 10;11:16176. doi: 10.1038/s41598-021-95508-3 (PMC8355112; doi:10.1038/s41598-021-95508-3)
Supplement: Supplementary file 2 — Supplementary Information 2. [file 41598_2021_95508_MOESM2_ESM.pptx]

## Slide 1
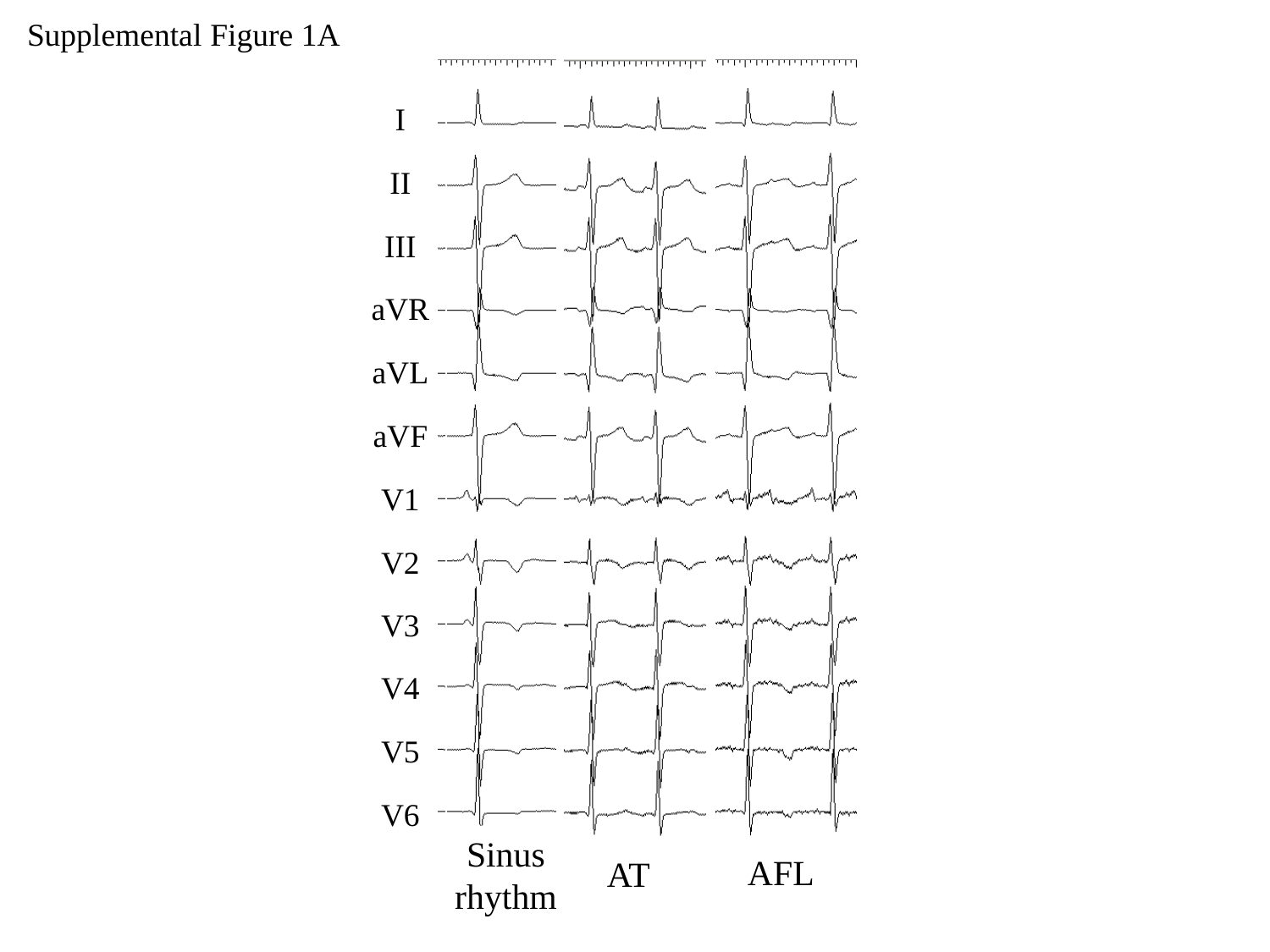

Supplemental Figure 1A
| I |
| --- |
| II |
| III |
| aVR |
| aVL |
| aVF |
| V1 |
| V2 |
| V3 |
| V4 |
| V5 |
| V6 |
Sinus rhythm
AFL
AT

## Slide 2
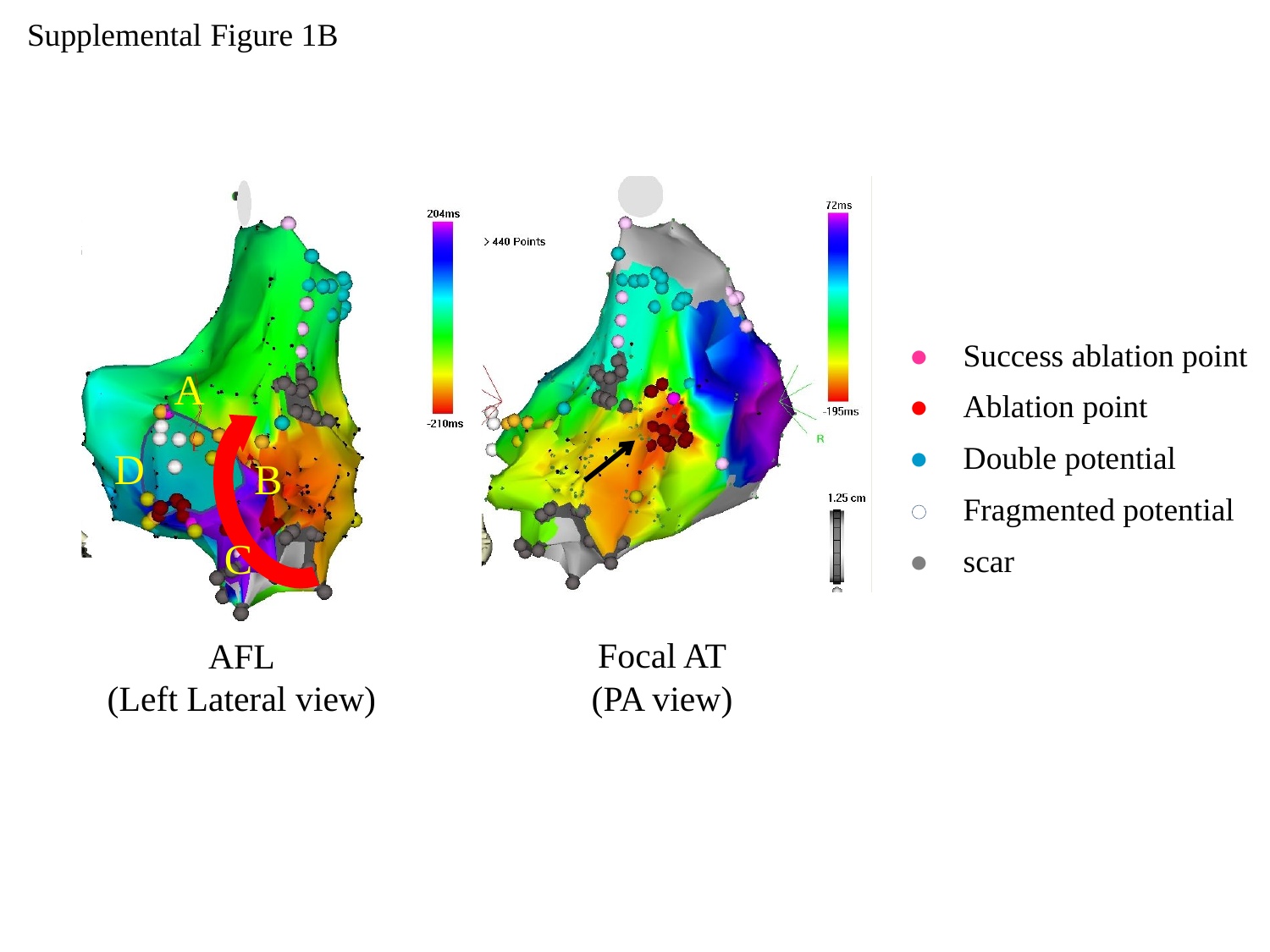

Supplemental Figure 1B
| ● | Success ablation point |
| --- | --- |
| ● | Ablation point |
| ● | Double potential |
| ● | Fragmented potential |
| ● | scar |
A
D
B
C
Focal AT
(PA view)
AFL
(Left Lateral view)

## Slide 3
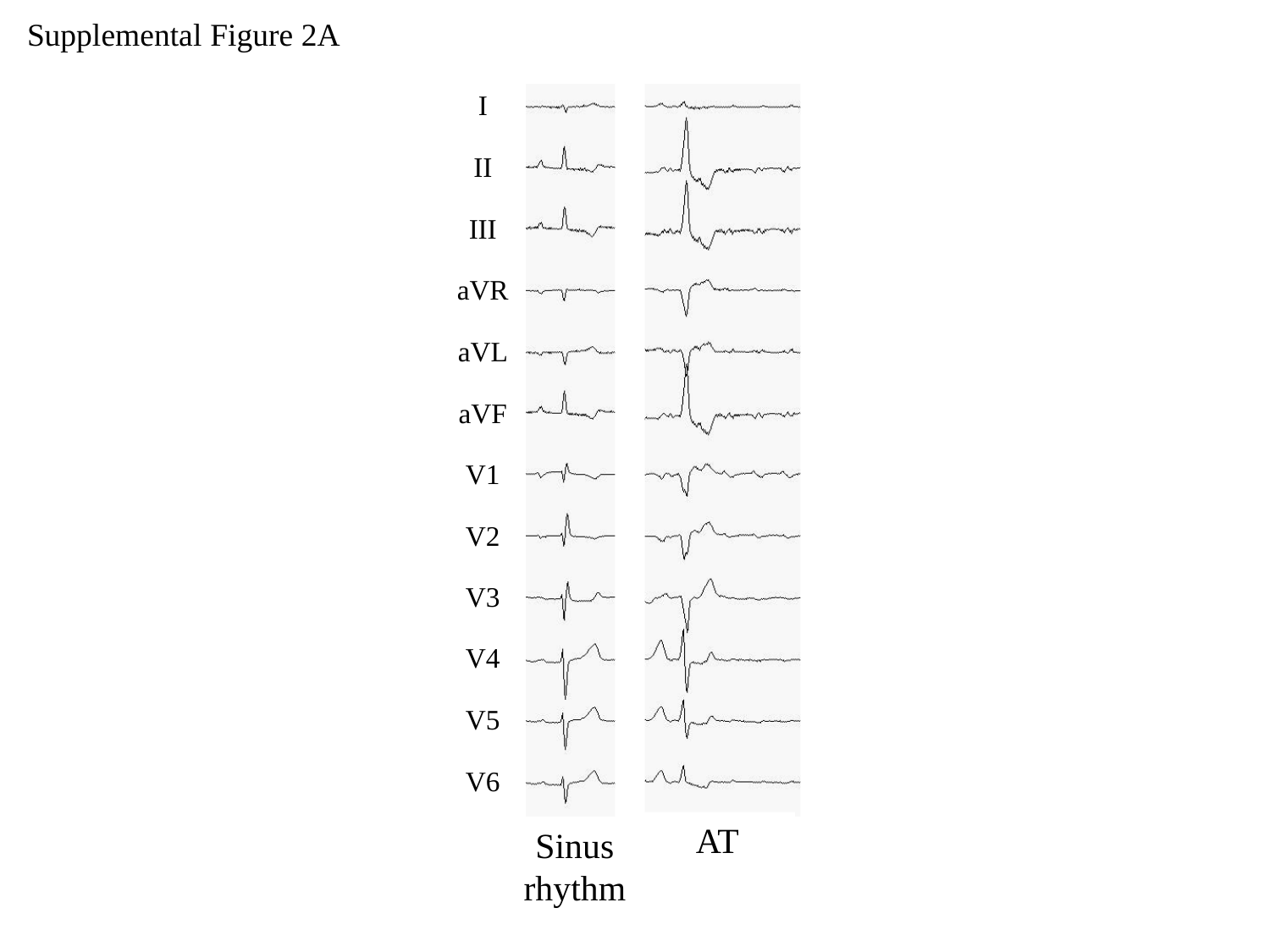

Supplemental Figure 2A
| I |
| --- |
| II |
| III |
| aVR |
| aVL |
| aVF |
| V1 |
| V2 |
| V3 |
| V4 |
| V5 |
| V6 |
AT
Sinus rhythm

## Slide 4
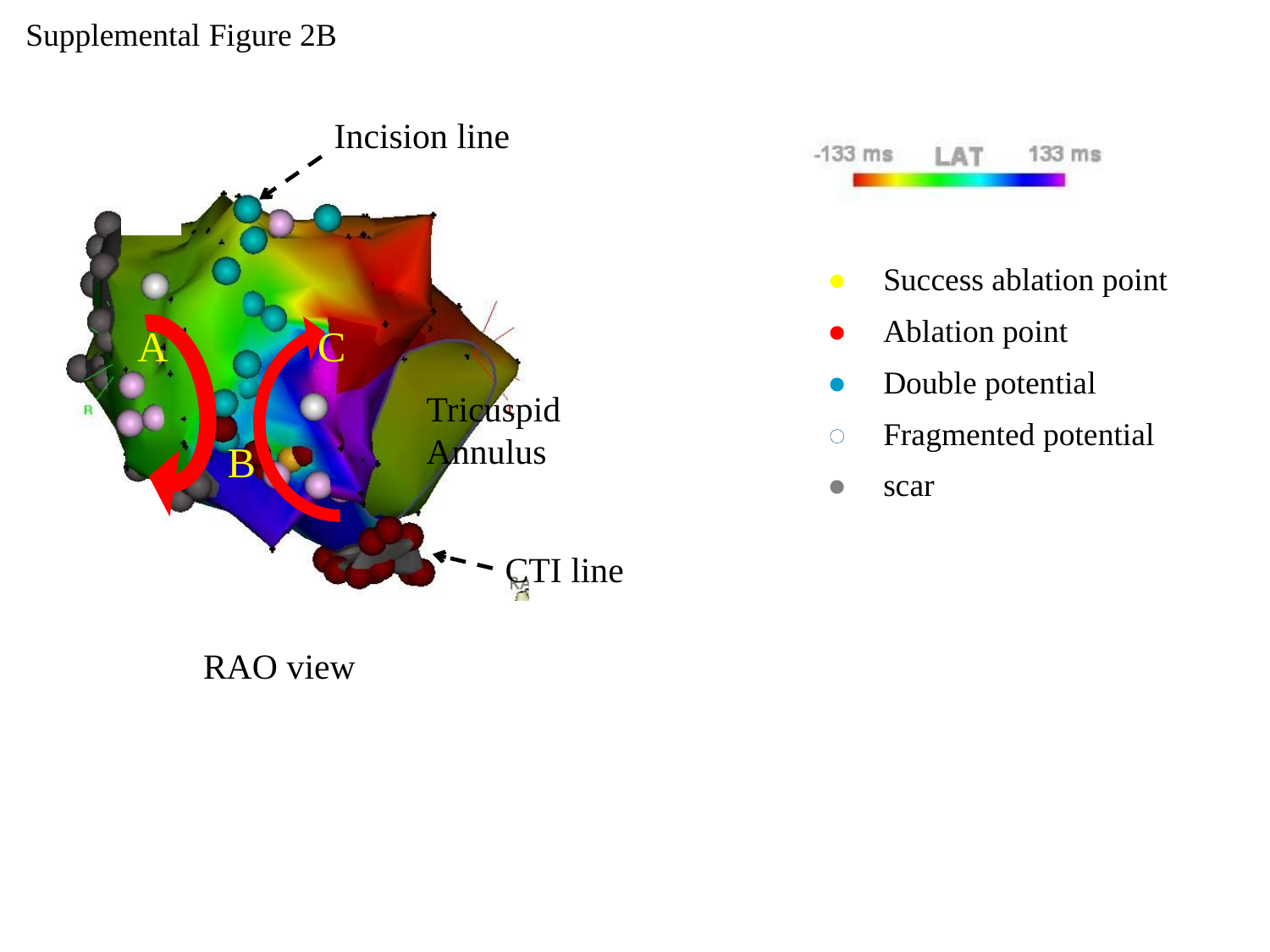

Supplemental Figure 2B
Incision line
| ● | Success ablation point |
| --- | --- |
| ● | Ablation point |
| ● | Double potential |
| ● | Fragmented potential |
| ● | scar |
A
C
Tricuspid
Annulus
B
CTI line
RAO view

## Slide 5
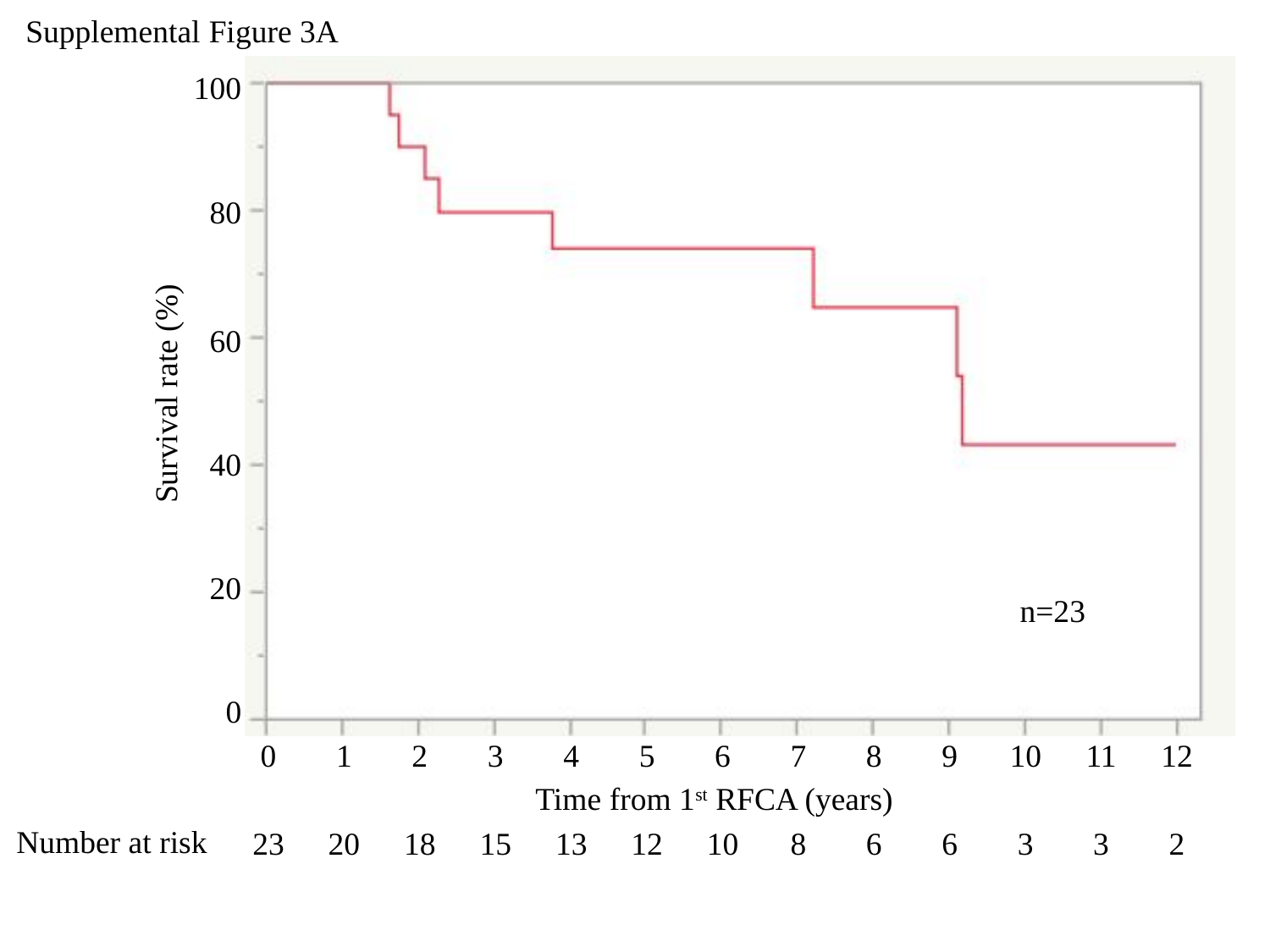

Supplemental Figure 3A
100
80
Survival rate (%)
60
40
20
n=23
0
| 0 | 1 | 2 | 3 | 4 | 5 | 6 | 7 | 8 | 9 | 10 | 11 | 12 |
| --- | --- | --- | --- | --- | --- | --- | --- | --- | --- | --- | --- | --- |
Time from 1st RFCA (years)
Number at risk
| 23 | 20 | 18 | 15 | 13 | 12 | 10 | 8 | 6 | 6 | 3 | 3 | 2 |
| --- | --- | --- | --- | --- | --- | --- | --- | --- | --- | --- | --- | --- |

## Slide 6
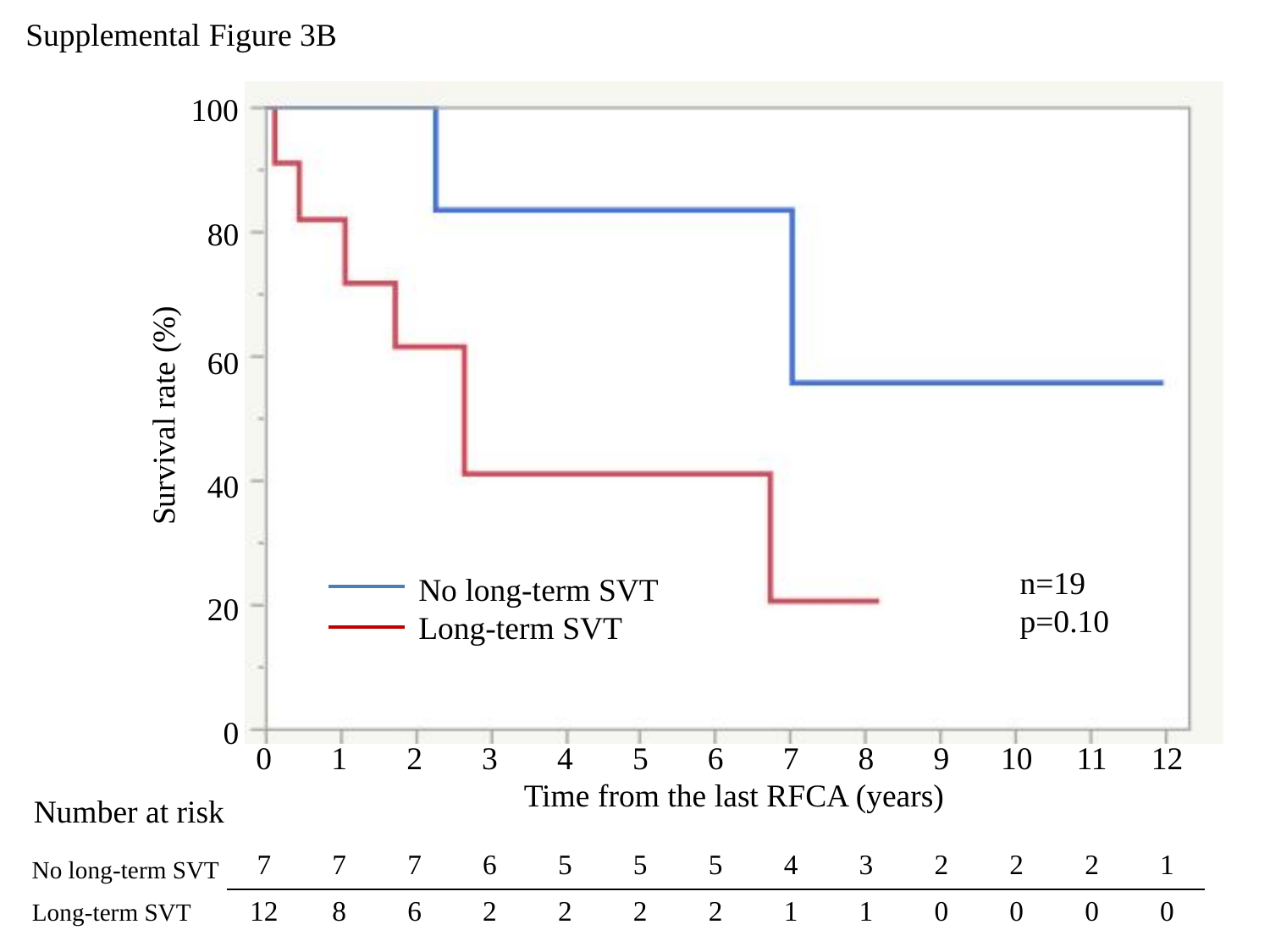

Supplemental Figure 3B
100
80
Survival rate (%)
60
40
n=19
p=0.10
No long-term SVT
Long-term SVT
20
0
| 0 | 1 | 2 | 3 | 4 | 5 | 6 | 7 | 8 | 9 | 10 | 11 | 12 |
| --- | --- | --- | --- | --- | --- | --- | --- | --- | --- | --- | --- | --- |
Time from the last RFCA (years)
Number at risk
| 7 | 7 | 7 | 6 | 5 | 5 | 5 | 4 | 3 | 2 | 2 | 2 | 1 |
| --- | --- | --- | --- | --- | --- | --- | --- | --- | --- | --- | --- | --- |
| 12 | 8 | 6 | 2 | 2 | 2 | 2 | 1 | 1 | 0 | 0 | 0 | 0 |
No long-term SVT
Long-term SVT
